# Risk taking under stress: the role(s) of self-selection. A comment on Buckert et al. (2014)

**DOI:** 10.3389/fnins.2014.00197

**Published:** 2014-07-14

**Authors:** Stefan T. Trautmann

**Affiliations:** Alfred-Weber-Institute for Economics, University of HeidelbergHeidelberg, Germany

**Keywords:** risk, ambiguity, stress, time pressure, self-selection

Observing behavior under extreme conditions (i) provides insights into decision making processes; and (ii) improves the external validity of predictions for behavior outside the lab, if the extreme condition is similar to a typical decision situation “in the field.” In terms of the first contribution, the study by Buckert et al. (2014) identifies a link between stress, cortisol levels, and risk attitude, supporting the view that hormone levels are an important moderator of risk taking and thus questioning the concept of stable risk preferences. However, no effect is found for ambiguity attitudes, suggesting that risk and ambiguity attitudes derive from different physiological and psychological processes (cf. Huettel et al., 2006; Levy et al., 2010). Regarding the second contribution, risky decision making under stress is typical for many professions, including such diverse occupations as medical doctors, police officers, or traders in financial markets. Policy and regulation thus need to take account of insights regarding the link between stress and risk taking to appropriately predict and regulate behavior in these professions.

While these insights are of broad interest, they should be taken with care. The interpretation of both contributions is potentially affected by selection problems that did not receive much attention in the study by Buckert et al. Regarding the physiological foundations of risk taking, Buckert et al. find that stress induction increases risk seeking, but only for those for whom the stress induction affected cortisol levels. Averaging over all participants, no effect was found. Indeed, it is clear that various potential mechanisms can exists that lead to a correlation of risk attitude and susceptibility to stress symptoms. Individuals' demographics, personality traits, and self-regulatory strategies have been shown to mediate responses to stress (Hagger, 2009); these same factors have also been associated with individual differences in risk attitudes (e.g., Borghans et al., 2008, on personality and demographics; Bryant and Dunford, 2008, on regulatory focus). Buckert et al. acknowledge that unobserved factors might be at play here, rendering the current evidence correlational (p. 9, section “Limitations”). But then we need to ask just how useful randomized treatment-control experiments with extreme conditions are, if they deliver only correlational data.

These problems are not restricted to the current setting with a psychological stress priming inventory. In experiments where participants make decisions under time pressure (which is one source of stress that is mentioned by Buckert et al.), a failure to make a decision within the time constraint similarly leads to a self-selected loss of observations. It is therefore important to tests whether those respondents who sometimes violate the time constraint differ systematically from those who do not violate time constraints (Kocher et al., 2013): this requires careful robustness checks using relevant, i.e., with respect to the preference under consideration, individual-level background data. It is an important open question whether decision makers who are susceptible to stress in situations as used in Buckert et al. and related studies are indeed similar to those who are not susceptible, in terms of economic preferences (e.g., risk, ambiguity, or time preference). That is, an explicit study of the heterogeneity in stress susceptibility and preferences would be warranted. Buckert et al. provide tools that can be used to elucidate these questions.

With respect to the second contribution of Buckert et al.'s study, i.e., the prediction of risky choices in situations outside the lab where decision makers act under stress, selection is a significant problem as well. It is clear that people who are highly susceptible to stress symptoms will select into different professions than those who are not affected by stress. But then heterogeneity in stress susceptibility, and the correlation of susceptibility with economic preferences, becomes essential for predicting behavior of people who self-selected in these professions. It has also been shown that people with low socio-economic status may experience systematically higher stress levels (e.g., Baum et al., 1999). The link between stress and attitudes toward uncertainty is therefore potentially informative on risky behavior across different socio-economic groups (e.g., Beckert and Lutter, 2013). However, people are not randomly allocated to their socio-economic status, and self-selection that is correlated with economic preferences is important (Burks et al., 2009). That is, in a wide range of applications, the link between stress and economic preference will be affected by self-selection. To make externally valid predictions, we need to understand the heterogeneity underlying the link between stress and preference.

The study by Buckert et al. employs state-of-the-art preference elicitation and stress induction methods. Extending these methods to carefully crafted within-person designs that elicit both baseline and treatment data on preferences for each participant, thus providing the relevant comparison measures to control for stress susceptibility, will allow identifying the crucial heterogeneity in the stress-preference link. Inventories for mediators of stress susceptibility that were identified in the literature can be included to inform about the processes that prevent stress effects for the economic preference under consideration for some respondents, but not for others. From the perspective of external validity, as well as with a view on potential interventions, identification of malleable mediating factors such as coping strategies or regulatory focus may be of special interest here (Higgins, 1997; Struthers et al., 2000). These results can then be compared to a complementary approach where cortisol is directly administered to participants (cf. Kandasamy et al., 2014), allowing to investigate in more detail the pathway from stress, through cortisol, to economic preferences and behavior.

## Conflict of interest statement

The author declares that the research was conducted in the absence of any commercial or financial relationships that could be construed as a potential conflict of interest.
